# Osteoblastic Differentiation and Mitigation of the Inflammatory Response in Titanium Alloys Decorated with Oligopeptides

**DOI:** 10.3390/biomimetics10010058

**Published:** 2025-01-16

**Authors:** Aroa Álvarez-López, Raquel Tabraue-Rubio, Rafael Daza, Luis Colchero, Gustavo V. Guinea, Martine Cohen-Solal, José Pérez-Rigueiro, Daniel González-Nieto

**Affiliations:** 1 Center for Biomedical Technology, Universidad Politécnica de Madrid, Pozuelo de Alarcón, 28223 Madrid, Spain; aroa.alvarez@ctb.upm.es (A.Á.-L.); rafael.daza@upm.es (R.D.); luis.colchero@ctb.upm.es (L.C.); gustavovictor.guinea@ctb.upm.es (G.V.G.); 2 Departamento de Ciencia de Materiales, Escuela Técnica Superior de Ingenieros Caminos, Canales y Puertos, Universidad Politécnica de Madrid, 28040 Madrid, Spain; 3 Biomedical Research Networking Center in Bioengineering Biomaterials and Nanomedicine (CIBER-BBN), 28029 Madrid, Spain; 4 Instituto de Investigación Sanitaria del Hospital Clínico San Carlos (IdISSC), Calle Profesor Martín Lagos s/n, 28040 Madrid, Spain; 5 INSERM U1132 Bioscar, Université de Paris Cité, 75006 Paris, France; martine.cohen-solal@inserm.fr; 6 Department of Rheumatology, APHP, Lariboisière Hospital, 75010 Paris, France; 7 Departamento de Tecnología Fotónica y Bioingeniería, ETSI Telecomunicaciones, Universidad Politécnica de Madrid, 28040 Madrid, Spain

**Keywords:** biomaterials, R-THAB^®^ functionalization, titanium, peptides, calvarial model

## Abstract

Under benign conditions, bone tissue can regenerate itself without external intervention. However, this regenerative capacity can be compromised by various factors, most importantly related with the extent of the injury. Critical-sized defects, exceeding the body’s natural healing ability, demand the use of temporary or permanent devices like artificial joints or bone substitutes. While titanium is a widely used material for bone replacement, its integration into the body remains limited. This often leads to the progressive loosening of the implant and the need for revision surgeries, which are technically challenging, are commonly associated with high complication rates, and impose a significant economic burden. To enhance implant osseointegration, numerous studies have focused on the development of surface functionalization techniques to improve the response of the body to the implant. Yet, the challenge of achieving reliable and long-lasting prostheses persists. In this work, we address this challenge by applying a robust and versatile biofunctionalization process followed by the decoration of the material with oligopeptides. We immobilize four different peptides (RGD, CS-1, IKVAV, PHSRN) on R-THAB^®^ functionalized surfaces and find them to be highly stable in the long term. We also find that RGD is the best-performing peptide in in vitro cell cultures, enhancing adhesion, proliferation, and osteogenic differentiation of mesenchymal stem cells. To assess the in vivo effect of RGD-decorated Ti-6Al-4V implants, we develop a calvarial model in murine hosts. We find that the RGD-decoration remains stable for 1 week after the surgical procedure and reduces post-implantation macrophage-related inflammation. These results highlight the potential of peptide decoration on R-THAB^®^ functionalized surfaces to expedite the development of novel metallic biomaterials with enhanced biocompatibility properties, thereby advancing the field of regenerative medicine.

## 1. Introduction

Bone tissue is a dynamic structure that has the potential to regenerate and restore its biological and mechanical properties when facing a fracture [1]. Spontaneous bone regeneration combines both intramembranous ossification (direct bone formation) in the periphery of the fracture and endochondral ossification (through a cartilage intermediate) within the fracture [2]. This physiological ability to form bone spontaneously promotes the repair of small injuries without external contribution. However, this healing process can be influenced by several factors, including patient characteristics (age, chronic diseases, etc.), infections [3], and the severity and extension of the injury [4]. Chronic conditions such as diabetes or osteoporosis, or an advanced age, can negatively impact bone regeneration due to a depletion of essential cell lineages and to the disruption of normal bone homeostasis [2,5]. However, the severity of the bone fracture itself has a greater impact on the healing process. For instance, in long bones, a loss of bone tissue that exceeds twice the diaphysis diameter will not heal spontaneously, even in patients with no additional pathologies [6]. These types of fractures are called critical-sized bone defects, and they do not heal on their own within a patient’s lifetime, even when using additional stabilization methods (plates, screws, etc.) [6,7]. When spontaneous regeneration fails, surgical intervention and implantation of temporary or permanent devices, such as artificial unions or bone substitutes, becomes necessary [8,9].

Around 2.2 million orthopedic procedures involving bone grafting take place annually worldwide [10]. Traditional bone grafts are sourced from different origins: autografts from the patient [11], allografts from donors or cadavers, and xenografts from animals [12]. While providing excellent osteogenic potential [12], they are associated with several drawbacks, such as harvesting complications in the case of autografts or the risk of immune rejection and infection transmission in allografts and xenografts [13]. To bypass these limitations, the development of synthetic alternatives has garnered significant attention and extensive research. However, not all synthetic materials are suitable for biomedical applications, as they must meet stringent specifications regarding their mechanical and biological properties. To ensure optimal mechanical properties, an ideal biomaterial should exhibit the following characteristics: (i) low corrosion rate and high wear resistance, (ii) prolonged fatigue life, and, preferably, (iii) an elastic modulus comparable to that of bone. Regarding biological properties, implanted materials should not impede local or systemic biological processes, although it has been suggested that a controlled level of reactivity between the implant and the adjacent tissues can enhance the healing process [14,15]. In this context, autografts, allografts, biodegradable ceramics, or polymers are often selected for bone reconstruction applications as they can be gradually replaced by newly formed bone tissue over time [16,17]. However, for stabilization purposes (e.g., fracture fixation, spinal fusion, or arthrodesis) and permanent replacements (e.g., arthroplasties), metal-based structures are commonly employed because of their ability to support body weight [18,19]. Among these, titanium materials are preferred due to their elastic modulus, which is closer to that of bone compared with other metallic alternatives [9].

Despite their good biocompatibility and widespread use, implanted metallic prostheses are still recognized as “foreign bodies” by the host immune system. In this regard, the response of the immune system and the subsequent inflammatory process are key early elements for the future fate of the material in the organism [20,21]. Thus, upon implantation, the immune response triggers inflammatory cascades that result in fibrosis and collagen encapsulation [22]. This connective tissue capsule isolates the implant from the surrounding healthy tissue, hindering proper osseointegration and fixation to the bone. The lack of osseointegration can be further compromised by daily movement and exercise, leading to a progressive loss of mechanical fixation over time, which does not imply any infection or trauma [23]. This condition, known as aseptic loosening, remains a significant challenge since it is a major limitation for the in-service life of the implant [23]. Aseptic loosening can lead to pain, instability, and reduced quality of life, exacerbated by physical activity and weight-bearing [24]. Moreover, it requires revision surgeries that are technically demanding procedures with high complication rates and significant associated costs [24]. To improve patient outcomes and reduce the associated economic burden, researchers are focusing on understanding the mechanisms of aseptic loosening and developing strategies to prevent it.

Thus, it has been found that the specific reactions that occur on the surface of an implant determine the degree of inflammation, healing rate, and long-term performance [25]. Consequently, implants with poor bioactive surfaces increase failure rates and the risk of infection due to prolonged healing times [9]. Significant advancements in biomaterial development have been made to create more efficient scaffolds that promote functional tissue regeneration rather than fibrous tissue formation and the subsequent creation of the connective tissue capsule. Among the proposed strategies, surface functionalization is a field focused on modifying the surface properties of the biomaterial to achieve specific traits that can benefit a particular application. This can be achieved through various methods, including mechanical techniques like grinding or polishing to modify the surface roughness and morphology [26]. In particular, there seems to be a significant effect of the topography of the material on the response of the organism to the implant that influences the final outcome of the prosthesis [27,28]. Alternatively, the biomaterial can be modified through chemical methods like alkali treatments and pickling, which allow the creation of specific chemically modified surfaces [29,30,31]. More recently, a new approach known as “biological or biochemical functionalization” has emerged, offering the potential to directly control biological interactions between the implant and the body. These new methodologies combine surface modification techniques with crosslinking chemistries to introduce reactive groups onto the surface to which specific molecules with bioactive functions can be covalently bound.

Previous decoration procedures relied on full-length proteins covalently anchored to the surface of the biomaterials, proving the ability of the decorated material to increase cell adhesion to the surface [32]. However, due to limitations associated with using full-length proteins, subsequent studies [33] explored the possibility of using smaller extracellular matrix (ECM)-derived oligopeptides for the decoration of the biomaterial. Compared to full-length proteins, peptides offer several advantages: lower cost [34], increased resistance to denaturation [35,36], higher purity, and enhanced long-term stability and functionality [35]. These characteristics would be advantageous when applying the peptide-decorated Ti-6Al-4V materials in vivo, as the in vivo environment is far more complex and demanding than a simplified in vitro condition.

This rationale is followed in this work by immobilizing some selected extracellular matrix peptides on functionalized R-THAB^®^ Ti-6Al-4V substrates produced by Bioactive Surfaces S.L. (Madrid, Spain). The assessment of the decorated materials is performed at three levels: (i) the analysis of the feasibility of using peptides with different physicochemical properties for the decoration of the functionalized biomaterial, including the evaluation of the long-term (months) stability of the decoration; (ii) in vitro analysis of the biological performance of the decorated substrates as obtained from cell cultures of mesenchymal stem cells (MSC), including, significantly, the ability of these substrates to induce osteogenic differentiation; and (iii) in vivo study of the performance of the decorated substrates using a calvarial mouse model that illustrates their reduced inflammatory response when compared with the bare titanium implants.

## 2. Materials and Methods

The experimental procedures described in detail in Section 2 correspond to the three levels of the characterization performed on the decorated materials. In this regard, the experimental procedures related with the assessment of the decoration process include the labeling of the selected peptides and the description of the crosslinking chemistry employed for its subsequent immobilization on the functionalized substrate. The in vitro studies comprise the assessment of the adhesion, survival, and proliferation of mesenchymal stem cells on the decorated materials. Additionally, specific details are provided on the measurement of the adhesion of the MSCs to the substrates through single cell force spectroscopy (SCFS) and inverted centrifugation assays, and the culturing conditions that lead to the osteogenic differentiation of the MSCs on the material are highlighted. The last set of experimental procedures comprises the analysis of the in vivo performance of the materials as obtained from a calvarial mouse model. The surgical procedure is described in detail and the experiments used to characterize the stability of the decoration, as well as the initial inflammatory response to the material upon implantation, are presented.

### 2.1. Functionalized Substrates

R-THAB^®^ Ti-6Al-4V and glass substrates produced were kindly provided by the company Bioactive Surfaces S.L. (Madrid, Spain). Low-roughness materials (surface rms < 15 μm) were used in this study.

### 2.2. Peptide Labeling with FITC

To visualize the peptides after surface immobilization, RGD (full sequence GRGDSP, Sigma-Aldrich, Saint Louis, MO, USA, SCP0157), CS-1 (EILDV, Bachem, Bubendorf, Switzerland, 01-H-2592), IKVAV (Bachem, 4052600), and PHSRN (Abyntek, Zamudio, Spain, custom synthesis) were labeled with fluorescein 5(6)-isothiocyanate (FITC, Sigma-Aldrich) following the protocol described elsewhere [33]. Briefly, the peptides were incubated for 3 h at room temperature and protected from light with a solution of FITC in PBS (phosphate-buffered saline) (proportion 1:0.5). After incubation, Spectra/Por CE Dialysis Tubing (Cole-Parmer, Vernon Hills, IL, USA, molecular weight cut-off = 500 Da) was used to remove the non-bound FITC molecules. The dialysis was performed against PBS as medium, which was changed every 12 h. During each change, 1 mL of that PBS medium was analyzed using a spectrophotometer (HALO Rb-10) at a wavelength of 495 nm (FITC excitation peak). After 3 to 4 changes, when the absorbance of the medium reached a value inferior to 0.004, the dialysis was stopped. At this point, the concentration of FITC remaining inside the dialysis tube was considered negligible. The FITC-labeled peptides were stored at −20 °C until further use.

### 2.3. Covalent Immobilization of FITC-Labeled Peptides for Stability Assays

FITC-labeled peptides were covalently bound to R-THAB^®^ functionalized samples using the EDC/NHS crosslinking chemistry. Briefly, a solution of 500 µg/mL of the FITC-labeled peptide was diluted in 4-morpholine-ethanesulfonic acid (MES, Sigma-Aldrich; 0.3 M in distilled water, pH = 5.0–6.0) to reach an intermediate concentration of 266 µg/mL. Then, 75 µL of this solution were added to each sample and incubated for 1 h at room temperature. Subsequently, 25 µL of a solution of 0.5 mg/mL EDC (Sigma-Aldrich) and 0.126 mg/mL NHS (Sigma-Aldrich) diluted in MES buffer (0.1 M in distilled water, pH = 5.0–6.0) were added to the samples and the incubation was continued for 4 h more. The final concentrations of the reagents were FITC-labeled peptide = 200 µg/mL, MES = 0.1 M, EDC = 0.125 mg/mL, and NHS = 0.0315 mg/mL.

To evaluate the long-term stability of the FITC-labeled peptides, the peptide-decorated R-THAB^®^ samples were immersed in 10% sodium dodecyl sulfate (SDS, Sigma-Aldrich) in PBS for 24 h to remove the initial non-covalently bound peptides. After incubation, samples were cleaned by three 5 min immersions in PBS and distilled water, sequentially, and dried with an argon flow. Images of the FITC-labeled peptides immobilized on the surfaces (day 0) were taken using an inverted fluorescence microscope (Leica DMI 3000B, Leica, Wetzlar, Gemany). Afterwards, samples were maintained in PBS at 4 °C and protected from light for 2 and 5 months. After each timepoint, samples were cleaned using the same procedure performed on day 0 and images of the FITC-labeled peptides that remained immobilized on the Ti-6Al-4V surfaces were acquired.

The fluorescence intensity inferred from the FITC-labeled peptides was quantified from the microscope images using ImageJ 1.54 software (NIH, https://imagej.nih.gov/ij/; accessed on 30 September 2019) and normalized by the fluorescent signal of an FITC solution of known concentration prepared at every time point of the study. This way we could avoid in our measurements a possible influence of uncontrolled variations in the microscope light intensity between time points. In addition, as a control of fluorescence photobleaching, a solution of each peptide-FITC was kept under the same conditions as the R-THAB^®^ Ti-6Al-4V samples, and the fluorescence intensity was analyzed at the same time points. All the images in this study were taken using the same conditions (Software LAS V4.2, Leica, Wetzlar, Germany): exposure time = 1.3 ms, gain = 2.1×, and gamma = 0.68.

### 2.4. Isolation and Expansion of MSC

The effects of the peptide-decorated biomaterials on cell behavior were assessed with mesenchymal stem and progenitor cells (MSCs). MSCs were isolated as described elsewhere [37]. Concisely, stromal cells were seeded at a density of 6.5 × 10^5^ cells/cm^2^ on fibronectin-decorated plates (Corning) in Iscove Modified Dulbecco’s Medium (IMDM, HyClone, Danaher Corporation, Washington, DC, USA) supplemented with 20% of MSC stimulatory supplements (StemCell Technologies, Vancouver, BC, Canada), 100 μM 2-mercaptoethanol, 1% penicillin/streptomycin (Sigma-Aldrich), 2 mM L-glutamine (Fisher Scientific, Waltham, MA, USA), 10 ng/mL human platelet-derived growth factor (PDGF-BB, Prepotech, Waltham, MA, USA, 100-14B), and 10 ng/mL recombinant mouse epidermal growth factor (rM-EGF, Prepotech, 315-09). Adherent cell clusters were grown for a minimum of five passages. For the following steps, cells were maintained in DMEM (Gibco, Waltham, MA, USA) supplemented with 10% fetal bovine serum (FBS, HyClone), 1% penicillin/streptomycin, and 1% L-glutamine. The experiments were performed with cells in passage between 6 and 15.

### 2.5. Covalent Immobilization of Peptides on R-THAB^®^ Functionalized Biomaterials for Cell Culture Assays

For cell studies, the immobilization process was the same as described in Section 2.4, but using unlabeled (non-fluorescent) peptides and a more stringent cleaning process to reduce a possible cytotoxic effect of free cross-linker molecules that could remain in the samples. Therefore, after completing peptide immobilization, samples were rinsed with distilled water and cleaned twice by a 2 h immersion in PBS. Subsequently, samples were immersed in MES 0.1 M for 72 h and sterilized using UV irradiation for 20 min on each side. Finally, the R-THAB^®^ Ti-6Al-4V substrates were immersed in Dulbecco’s Modified Eagle Medium (DMEM, Gibco, pH  =  7.4) twice for 12 h each time. The cleaning procedure was performed under sterile conditions, and during the different incubation steps, samples were kept at 4 °C.

### 2.6. Cell Adhesion Assay

To characterize the adhesion kinetics of MSCs on IKVAV- or PHSRN-decorated R-THAB^®^ Ti-6Al-4V samples, cells were seeded at a concentration of 10 × 10^4^ cells/mL (500 µL per well) on each substrate, previously placed on 24-well plates (Fisher Scientific). Incubation was performed for 2 and 6 h at 37 °C in a humidified atmosphere of 5% CO_2_. After each timepoint, cells were stained with Calcein AM (0.5 μM in dimethyl sulfoxide (DMSO), Fisher Scientific) for 15 min at 37 °C. After incubation, samples were cleaned with PBS and observed using a fluorescence microscope. At least five representative images were taken from each replica per timepoint. To quantify the number of attached cells per substrate, the microscope images were manually counted using ImageJ and the sum of cells per field was extrapolated to the number of cells per cm^2^ using the image scale (objective magnification 10×, total magnification 100×).

### 2.7. Single Cell Force Spectroscopy (SCFS) Assay

To quantify the strength of adhesion of single cells to peptide-decorated R-THAB^®^ glass surfaces, SCFS assays were performed using an atomic force microscope (AFM). The main guidelines to perform this procedure were already described elsewhere [38]. A Nanolife AFM microscope (Nanotec Electrónica, Madrid, Spain) coupled to an inverted light microscope was used to perform these assays. After its calibration, a cantilever previously decorated with concanavalin A was immersed in PBS for approximately one hour at room temperature (25 °C) to ensure thermal equilibrium and reduce thermal drift. The concanavalin A-decorated cantilever was obtained after the following sequential incubations: 0.5 mg/mL biotin-BSA (Sigma-Aldrich) in sodium hydrogen carbonate at 37 °C overnight, 0.5 mg/mL streptavidin (Sigma-Aldrich) in PBS for 30 min at room temperature, and 0.4 mg/mL concanavalin A biotinylated (Sigma-Aldrich) in PBS for 30 min at room temperature.

To prepare the cells used for this assay, MSCs were detached from the growing plates using Trypsin-EDTA 0.05% and counted. After placing the peptide-decorated glasses on the AFM sample holder, 2 mL of filtered complete growth medium was added to cover the samples and 2 × 10^3^ cells were transferred from the MSC suspension to perform the assay. After attaching a single cell to the tip of the cantilever, the assay consisted of a series of approach–contact–retraction cycles between that cell and the peptide-decorated glass surfaces. The specific parameters used in this article were retraction speed = 37 µm/s and contact time = 15 s. To quantify the detachment force between the cell and the decorated surface, a MATLAB routine was used to calculate the difference between baseline deflection and the deepest peak registered during retraction.

### 2.8. Inverse Centrifugation Assay

To quantify the adhesion strength of the MSC population to peptide-decorated R-THAB^®^ glass samples, inverse centrifugation assays were performed as described elsewhere [38]. Cells were seeded at a density of 50 × 10^3^ cells/mL (500 µL per well) on each substrate, previously placed on 24-well plates. Incubation was performed for 24 h at 37 °C in a humidified atmosphere of 5% CO_2_. The next day, cells were stained with Calcein AM (0.5 μM in DMSO) for 15 min at 37 °C. After incubation, samples were cleaned with PBS and observed using a fluorescence microscope (input). Subsequently, glass samples were placed upside down in PBS-filled Eppendorf tubes and secured with a sealing film. Centrifugation was conducted for 5 min at 1, 2, and 8 relative centrifugal force (RCF, Hettich Rotina 380 R centrifuge. Aizarnazabal, Spain). After centrifugation, fluorescence microscope images of the remaining cells were taken (output). In both cases, before and after treatment, at least five representative images were taken from each replica per condition. To quantify the percentage of attached cells (100 × (output/input)) after each centrifugation step, microscope images were manually counted using ImageJ. The sum of cells per field was extrapolated to the number of cells per cm^2^ using the image scale (objective magnification 10×, total magnification 100×). As positive controls for this assay, bare borosilicate coverslips were incubated with 4 µg/cm^2^ of fibronectin (Sigma-Aldrich, F4759) in PBS for 45 min at 37 °C.

### 2.9. Proliferation and Survival Assay

To characterize the proliferation and survival rate of MSCs on bare and R-THAB^®^ Ti-6Al-4V substrates, 6 × 10^3^ cells/mL (500 µL per well) were seeded on each substrate, previously placed on 24-well plates. Incubation was performed for 3, 7, 10, and 20 days at 37 °C in a humidified atmosphere of 5% CO_2_. The survival analysis (>day 10) was performed replacing the growth medium (10% FBS) by a 1% FBS medium to reduce cell metabolism and prevent detachment due to excessive cell growth. After each timepoint, cells were stained with a mixed solution of 1 µL/mL of calcein AM (0.5 μM in DMSO) and 1 µL/mL of propidium iodide (PI, 750 µM in PBS, Fisher Scientific, Waltham, MA, USA) for 15 min at 37 °C. After incubation, cells were observed under a fluorescence microscope and at least five representative images were taken from each replica per timepoint. Images were manually counted using ImageJ and the sum of cells per field was extrapolated to the number of cells per cm^2^ using the image scale (objective magnification 10×, total magnification 100×). For the survival analysis, the percentage of calcein-positive cells was normalized by the total number of cells.

### 2.10. Osteoblastic Differentiation Assay

For the osteoblastic differentiation assay, 50 × 10^3^ cells/mL (500 µL per well) were seeded both on bare and on R-THAB^®^ functionalized Ti-6Al-4V substrates previously placed on 24-well plates. Incubation was performed at 37 °C in a humidified atmosphere of 5% CO_2_. When MSCs reached 80% confluence, the growth medium was exchanged for the osteogenic stimulatory medium (StemCell Technologies, 05504) and cells were incubated for 20 days. Hemi-depletions of the medium were performed every three days to ensure the accumulation of differentiation factors.

The expression level of alkaline phosphatase (ALP) was characterized by immunofluorescence analysis. After 20 days of incubation, cells were cleaned with PBS and fixed in 4% PFA in PBS for 15 min. After three 5 min PBS washing steps, cells were permeabilized using 0.1% Triton X-100 in PBS for 10 min. Subsequently, cells were cleaned by three 5 min PBS washing steps, and blocking was performed with 2% bovine serum albumin (BSA) in PBS for 1 h at room temperature. Afterwards, cells were labeled overnight using the primary polyclonal antibody anti-ALP made in rabbit (Fisher Scientific, 15934454) diluted 1:200 in 0.1% BSA in PBS. After overnight incubation, samples were cleaned with three 5 min PBS washing steps and incubated with the secondary antibody anti-rabbit Alexa Fluor^®^ 488 (Fisher Scientific, 10729174) diluted 1:500 for 4 h at room temperature. Subsequently, samples were cleaned with three 5 min PBS washing steps and the nucleus was counterstained using a solution of 0.5 μg/mL Hoechst 33258 in PBS for 5 min. To compare the intensity of ALP expression between samples, the corrected total cell fluorescence (CTCF) was quantified. First, at least five representative images were taken per replica using the same parameters (exposure time = 300 ms, gain = 4X, and gamma = 1). From each image, the integrated density and cell area values were calculated from 10 random cells and a background intensity value was obtained. Finally, CTCF was calculated using the following formula (CTCF = Integrated Density − (Cell area × background intensity)).

### 2.11. Animals

The in vivo studies were performed using 4- to 5-month-old CD1 wild type (30–35 g) male mice. These animals were raised and stored in the Centro de Tecnología Biomédica animal facility (registration number: ES280790002070). All mice had free access to food and water and were kept in rooms with controlled temperature, humidity, and light–dark cycle. All the procedures performed in this work were approved by the corresponding Ethic Committees (PROEX authorization number: 010.3/22) and were developed following the Spanish animal experimentation legislation (Real Decreto 53/2013 and Orden ECC/566/2015).

### 2.12. Surgical Procedure for Ti-6Al-4V Implantation—Calvarial Model

Circular R-THAB^®^ Ti-6Al-4V samples (3 mm diameter; 200 µm thickness) were kindly provided by Bioactive Surfaces S.L. for the in vivo assays. The 3 mm diameter implants were glued to 5 mm diameter circular borosilicate cover glasses (VWR) using a biocompatible silicon adhesive (Dow Chemical Company, DOWSIL™ 3140 RTV). This way, the implants could be easily fixed to the skulls.

To perform the surgeries, 0.1 mg/kg of buprenorphine (BUPREX^®^, Hull, UK, 0.3 mg/mL) was administered to each mouse by intraperitoneal injection before surgery (pre-surgery analgesics) and 5 times per animal every 12 h after surgery (post-surgery analgesics). The whole calvarial model procedure was performed under general anesthesia by the administration of inhaled isoflurane (2% in air). To maintain sterility, the hair on the mice’s heads was completely shaved and the skin was cleaned with 70% ethanol in PBS. Subsequently, small incisions were made on the skin, leaving the animal’s cranium exposed. Then, 3 mm craniotomies were made on the mice skulls using a drill (Dremel, Racine, WI, USA) with a circular hole saw drill bit, trying not to damage the dura mater and brain. After making the defects, the grafts (Ti-6Al-4V + cover glass) were glued to the craniotomies’ adjacent bone using a quick-drying glue (Henkel Adhesives, Loctite^®^ 454, Düsseldorf, Germany). To complete the procedure, the skin was sewed using 3-0 suture thread (Laboratorio Aragó, Barcelona, Spain).

### 2.13. In Vivo Stability of the Immobilized Peptide Layer on R-THAB^®^ Ti-6Al-4V

To study the in vivo stability of the immobilized peptides, the 3 mm inserts were decorated with the FITC-labeled RGD as described in Section 2.2 and Section 2.4. Before implantation, the (RGD-FITC)-decorated implants were observed under the fluorescence microscope to obtain the initial value of fluorescence intensity (day 0). Surgeries were performed following the procedure described in Section 2.12. On days 1 and 7 after implantation, mice were sacrificed by cervical dislocation and the implants were removed from the skulls. Images of the (RGD-FITC)-decorated implants were obtained using the fluorescence microscope and quantified using ImageJ. The fluorescence intensity of the implants of each timepoint (1 and 7) was normalized by the initial intensity before the surgical procedure (day 0). The samples were also normalized by the fluorescent signal of a FITC solution of known concentration prepared at every time point of the study. At least five representative images were taken per replica under the same conditions (Software LAS V4.2): exposure time = 1.3 ms, gain = 2.1X, and gamma = 0.68.

### 2.14. Characterization of the In Vivo Inflammatory Response After Decorated R-THAB^®^ Ti-6Al-4V Implantation

RGD-decorated implants were prepared following the standard procedures described in Section 2.2 and Section 2.5, and surgeries were performed following the method described in Section 2.12. On day 2 after implantation, mice were sacrificed by cervical dislocation and the implants were removed from the skulls. The implants were fixed in 4% PFA in PBS for 15 min and an immunofluorescence analysis was performed as described in Section 2.11. Ti-6Al-4V implants were labeled with the primary antibodies rabbit anti-Iba1 (1:1000, Abcam, ab178847) and rat anti-CD86 (1:100; Tebubio, 281MB65837, Barcelona, Spain), which label macrophagic populations. Primary antibodies were detected after labeling with the secondary antibodies anti-rabbit Cy3 (1:800; Jackson Immunoresearch, 111-165-003, Baltimore Pike, PA, USA) and anti-rat Alexa Fluor^®^ 488 (1:800; Jackson Immunoresearch, 712-546-153). Samples were observed under the fluorescence microscope, and at least five representative images per sample were taken. Iba1/CD86-positive or -negative populations were manually counted using ImageJ.

### 2.15. Statistical Analysis

Statistical analyses were performed with SigmaPlot (Systat, Chicago, IL, USA). Data are expressed as the means ± standard errors of the means (SEM). Significant differences between groups were determined using parametrical and non-parametrical statistical models, which are noted in each figure’s legend. In all cases, the results obtained from the tests were considered significant for *p*-values below 0.05, displayed as asterisks in the graphs.

## 3. Results

### 3.1. Long-Term Stability of (Peptide-FITC)-Decorated R-THAB^®^ Ti-6Al-4V

**Figure 1:**
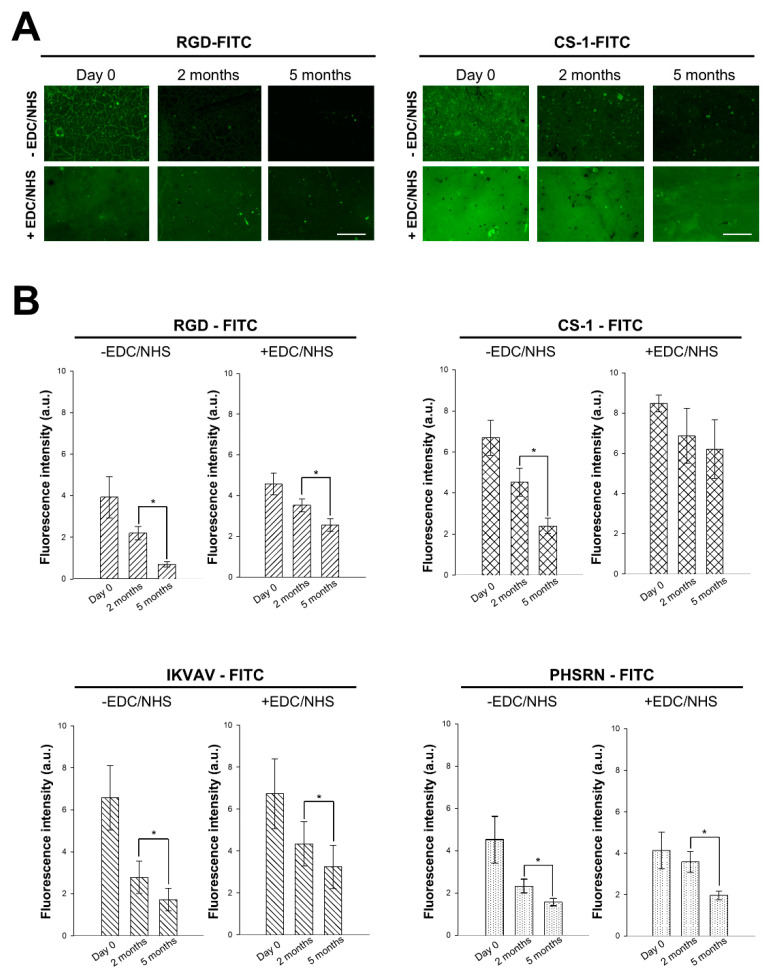
Long-term stability of FITC-labeled peptides immobilized on R-THAB^®^ Ti-6Al-4V substrates through EDC/NHS chemistry. (**A**) Representative fluorescence microscopy images of RGD-FITC and CS-1-FITC incubated on R-THAB^®^ Ti-6Al-4V without EDC/NHS (-EDC/NHS) or with EDC/NHS (+EDC/NHS). Samples are shown on day 0, 2 months, and 5 months after immobilization (scale bar = 270 μm). (**B**) Quantification of the mean fluorescence intensity measured from the attachment of each oligopeptide without EDC/NHS or with EDC/NHS on day 0 and after 2 and 5 months. The values are normalized to the background intensity and adjusted depending on the variations on the intensity of the microscope between time points. The data are shown as the mean ± SEM of at least 6 replicas per condition from two independent assays. The asterisks indicate significant differences between time points (paired *t*-test; one asterisk, *p* < 0.05).

As bone regeneration in vivo normally takes a minimum of 3 to 4 weeks [2,39], it was necessary to conduct assessment of the long-term stability of the decorated samples. Figure 1 compares the initial fluorescence observed in the decorated samples with that recorded 2 and 5 months after the immobilization process. It was observed that after 2 months, the fluorescence intensity of all four peptides remained relatively stable (Figure 1B). However, in the fifth month, we observed a significant drop in the fluorescence for all the samples, including those incubated with crosslinkers (+EDC/NHS). Among the decorated samples, CS-1-FITC was the most stable, while PHSRN-FITC displayed the highest fluorescence drop. Despite not being capable of extrapolating these results to the most complex in vivo condition, we concluded that a significant fraction of the immobilized peptides remained active even after 5 months of the immobilization process.

### 3.2. Cell Adhesion Studies on IKVAV and PHSRN-Decorated R-THAB^®^ Ti-6Al-4V

**Figure 2:**
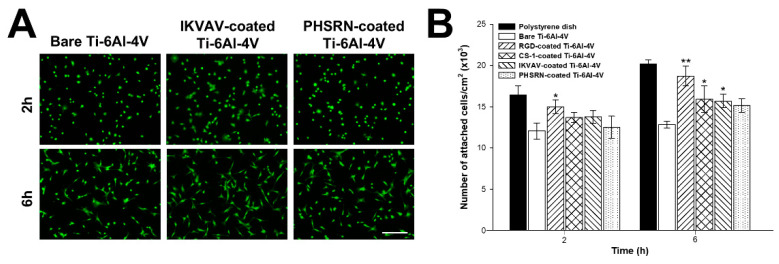
Adhesion analysis of MSCs seeded on polystyrene, bare Ti-6Al-4V and peptide-decorated R-THAB^®^ Ti-6Al-4V. (**A**) Representative fluorescence microscopy images of calcein-positive MSCs cultured on polystyrene, bare Ti-6Al-4V, IKVAV-decorated Ti-6Al-4V, and PHSRN-decorated Ti-6Al-4V at 2 and 6 h after seeding (scale bar = 200 μm). (**B**) Number of attached cells/cm^2^ at each time point. Data are shown as the mean ± the SEM of 6 replicas per temporal point and type of substrate from two independent assays. The asterisks denote significant differences between bare Ti-6Al-4V and each peptide-decorated R-THAB^®^ Ti-6Al-4V (two-way ANOVA followed by Tukey’s test; one asterisk, *p* < 0.05; two asterisks, *p* < 0.01).

To check the effect of each peptide on cell adhesion, we first determined the number of cells attached to the different substrates (control polystyrene, control bare Ti-6Al-4V, and decorated R-THAB^®^ Ti-6Al-4V) over the first hours after seeding, as shown in Figure 2. It is observed that the number of cells attached to any of the peptide-decorated Ti-6Al-4V samples after 6 h of seeding is higher than on bare Ti-6Al-4V (Figure 2B). As discussed above, the usage of peptides has some advantages in terms of the control on the experimental system and, correspondingly, in terms of reproducibility, at the expense of a reduction in the cues offered for the interaction between the biomolecule and the cell. In this regard, and although no controls are included here that compare the performance of the peptides with the full proteins, such a comparison may rely on previously published works on protein-decorated samples employing either collagen or fibronectin [32,40], where similar results to those included in this work were reported.

### 3.3. Single Cell Force Spectroscopy on Peptide-Decorated R-THAB^®^ Glass

**Figure 3:**
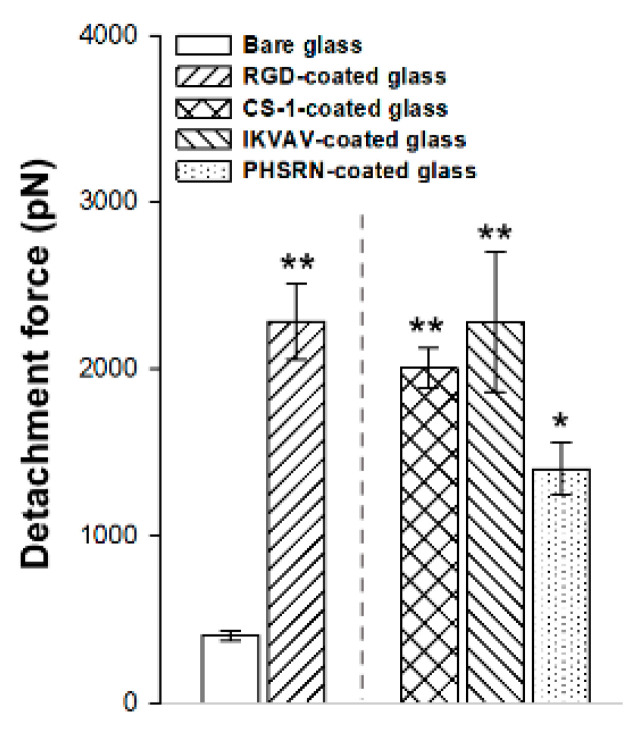
Single cell force spectroscopy (SCFS) assay on peptide-decorated R-THAB^®^ glasses displaying the detachment force values between the cell and peptide-decorated or the bare glasses. Data are shown as mean value ± SEM. The asterisks indicate significant differences in cell adhesion between bare glass and each peptide-decorated glass (one-way ANOVA followed by Tukey’s test; one asterisk, *p* < 0.05; two asterisks, *p* < 0.01).

SCFS allows accurately measuring the adhesion force of a single cell to a substrate. The detachment force values obtained after analyzing the AFM force–distance curves from each peptide are gathered in Figure 3. RGD-decorated and IKVAV-decorated surfaces displayed the highest values, while PHSRN-decorated surfaces showed the lowest ones. It can be observed how the adhesion force of the cell to the substrate is larger for any of the peptide-decorated samples than for the bare glass (control) sample, although some differences are found between the peptides that can be assigned to their distinct sequences.

### 3.4. Inverted Centrifugation Analysis on Peptide-Decorated R-THAB^®^ Glass

**Figure 4:**
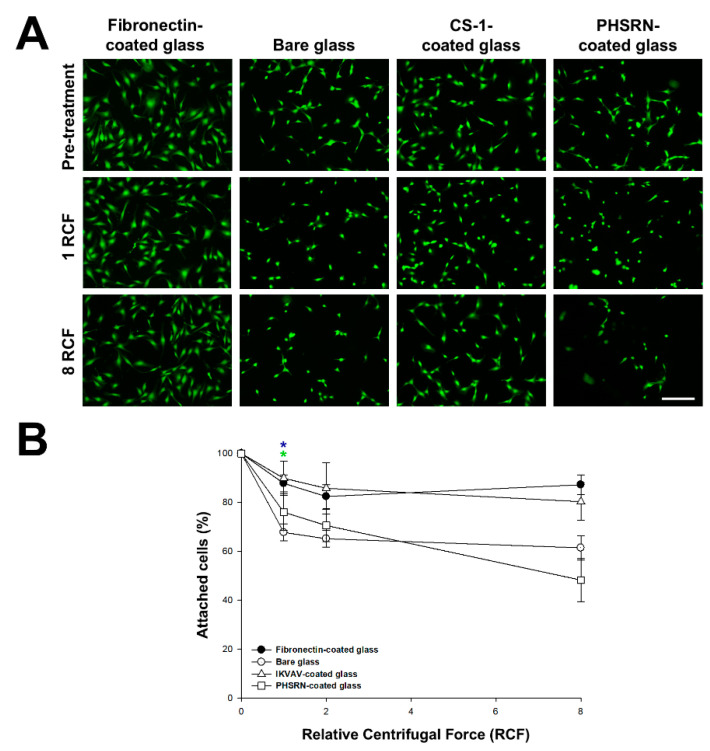
Inverted centrifugation assays of MSCs after 1 day of seeding on fibronectin-decorated glass, bare glass, and peptide-decorated R-THAB^®^ glasses. (**A**) Fluorescence microscope images of calcein-positive MSCs cultured on fibronectin-decorated glass, bare glass, CS-1-decorated R-THAB^®^ glass, and PHSRN-decorated R-THAB^®^ glass before and after centrifugation (scale bar = 250 μm). (**B**) Percentage of cells still attached to the different surfaces after every centrifugation step (1, 2, or 8 RCF). Data are displayed as the means ± the SEM with at least 6 replicas from every condition obtained in two independent experiments. The blue and green asterisks denote statistically significant differences between bare and fibronectin coated glass, and between bare and IKVAV-decorated glass, respectively (two-way ANOVA followed by Tukey’s test; one asterisk, *p* < 0.05).

To complement the measurements of the initial adhesion of the cells to the substrates obtained with the SCFS, inverted centrifugation studies were used (Figure 4). The quantitative results obtained for the CS-1-, IKVAV-, and PHSRN-decorated glasses are shown in Figure 4B. At the selected centrifugal forces, 80–90% of the cells seeded on the fibronectin- (positive control), RGD-, CS-1-, and IKVAV-decorated glasses remained attached to the surface, while only 60–70% of the initial cells remained on bare glass samples. However, PHSRN-decorated glass did not seem to provide any enhancement in cell adhesion strength compared to bare glass after longer seeding times.

Table 1 summarizes the performance of each peptide in the in vitro studies. RGD excelled in all the tests performed compared to the other peptides, while PHSRN did not lead to any significant improvement compared to the bare Ti-6Al-4V surface. Considering these results, we decided to choose the RGD to examine the long-term survival of mesenchymal stem cells attached to RGD-decorated R-THAB^®^ Ti-6Al-4V, as a durable cell survival is indispensable considering the time period (3–4 weeks) required for MSC differentiation to osteoblast bone-forming cells.

### 3.5. Proliferation and Survival Analysis of RGD-Decorated R-THAB^®^ Ti-6Al-4V

**Figure 5:**
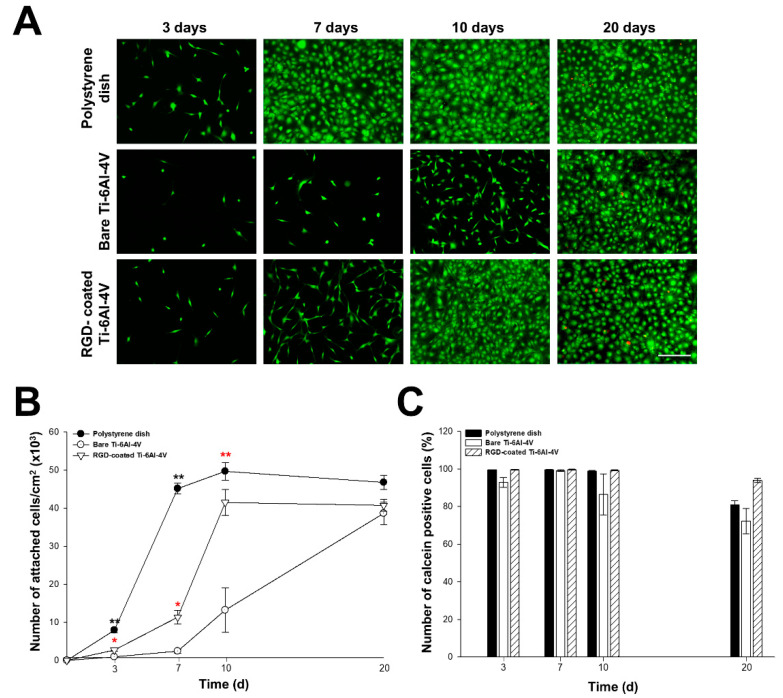
Proliferation and survival analysis of MSCs seeded on polystyrene, bare Ti-6Al-4V, and RGD-decorated R-THAB^®^ Ti-6Al-4V. (**A**) Representative fluorescence microscopy images of alive (calcein-positive—green) and dead cells (PI-positive—red) cultured on polystyrene, bare Ti-6Al-4V, and RGD-decorated Ti-6Al-4V for 3, 7, 10, and 20 days (scale bar = 240 μm). (**B**) Number of attached cells/cm^2^ per time point. (**C**) Percentage of calcein-positive (alive) cells per time point. Data are shown as the means ± the SEM of at least 6 replicas per temporal point and type of substrate from two independent experiments. The black asterisks denote significant differences between polystyrene and RGD-decorated Ti-6Al-4V. The red asterisks denote significant differences between bare and RGD-decorated Ti-6Al-4V (two-way ANOVA followed by Tukey’s test; one asterisk, *p* < 0.05; two asterisks, *p* < 0.01).

The results displayed in Figure 5 show that MSCs were able to proliferate on each substrate until reaching 100% confluence and survived for 20 days. However, RGD-decorated Ti-6Al-4V accomplished cell confluence significantly before bare Ti-6Al-4V (Figure 5A,B). The analysis of the proliferation rate between days 3 and 7 showed a proliferation deficit on bare Ti-6Al-4V, which was independent of the initial lower MSC adhesion that had been previously observed. In terms of survival rate (Figure 5C), the analysis of the percentage of calcein-positive cells throughout the study did not show any differences between substrates. Lower calcein-positive ratios were obtained on day 20, dropping in polystyrene and bare Ti-6Al-4V, but these differences were not statistically significant.

### 3.6. Osteoblastic Differentiation Analysis on RGD-Decorated R-THAB^®^ Ti-6Al-4V

**Figure 6:**
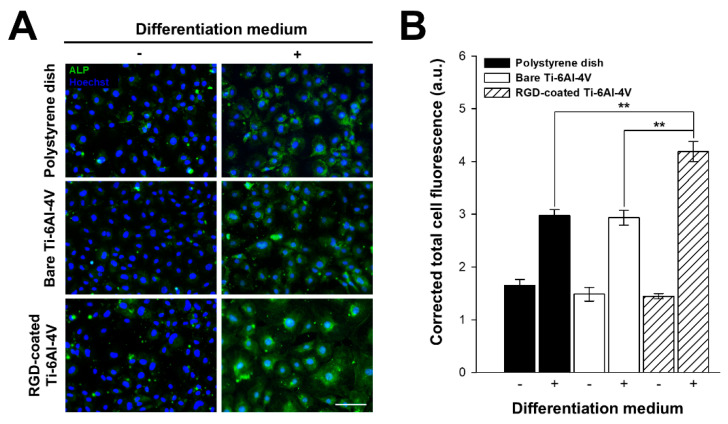
Osteoblastic differentiation assays performed on polystyrene, bare Ti-6Al-4V, and RGD-decorated R-THAB^®^ Ti-6Al-4V. (**A**) Representative fluorescence microscopy images of MSCs with or without osteoblastic differentiation medium on polystyrene, bare Ti-6Al-4V, and RGD-decorated Ti-6Al-4V. ALP expression is stained green and the nucleus blue (scale bar = 100 μm). (**B**) Quantification of the CTCF inferred from ALP expression in each analyzed cell. Data are shown as the mean ± the SEM of at least 230 cells from 6 replicas per substrate. The asterisks denote significant differences in ALP expression between substrates (Kruskal–Wallis test followed by Dunn’s method; two asterisks, *p* < 0.01).

We studied the in vitro osteoblastic differentiation capacity of our RGD-decorated Ti-6Al-4V, as it was an essential trait for its intended application. Figure 6 shows ALP expression in MSCs seeded on the different substrates. ALP expression increased when incubating the MSCs with the differentiation medium, as could be seen by the substantial rise on the CTCF, indicating a positive differentiation. Among the substrates, the CTCF intensity (Figure 6B) was higher in MSCs seeded on RGD-decorated Ti-6Al-4V, indicating an enhancement in differentiation promoted by the presence of the RGD peptide, even when compared with the polystyrene control. In contrast, no significant difference among the three substrates was observed when the cell culture proceeded in the absence of the differentiation medium.

### 3.7. Mouse Calvarial Model Surgical Procedure and RGD Layer Stability In Vivo

**Figure 7:**
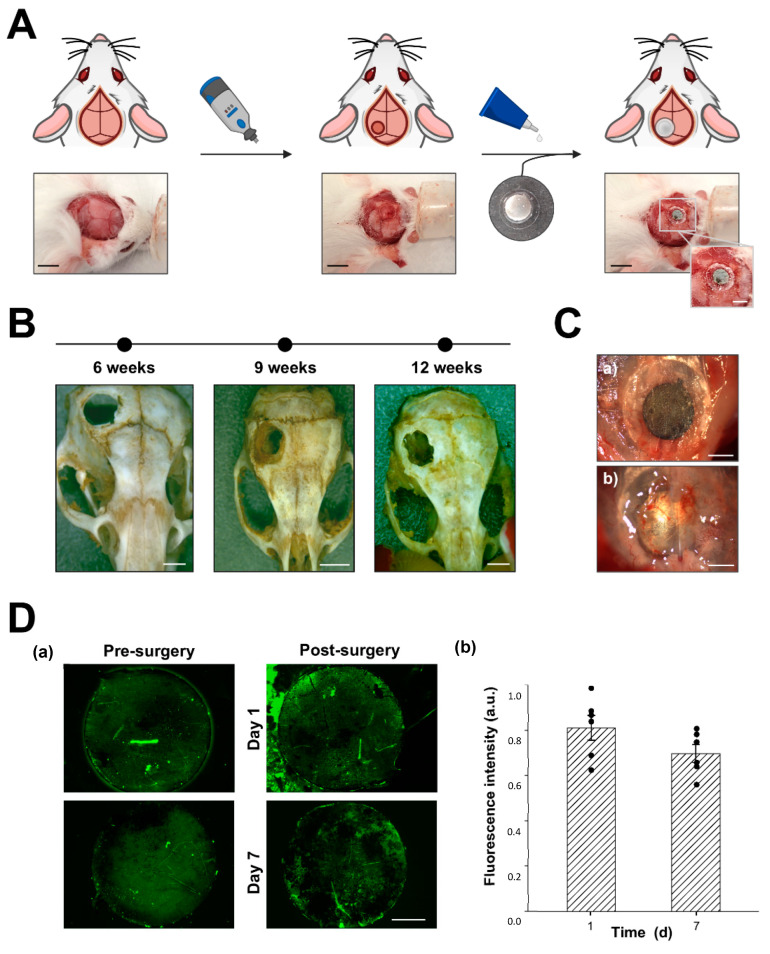
Calvarial model surgical procedure and in vivo stability of the (RGD-FITC)-decorated R-THAB^®^ Ti-6Al-4V. (**A**) Schematic representation of the two steps that comprise the surgical model: craniotomy preparation and graft implantation. (**B**) Representative photographs of mouse scalps with 3 mm craniotomies and their time evolution (scale bar = 3 mm). (**C**) Optical photographs taken with the stereo microscope of (**a**) the front side and (**b**) the inner side of the implanted graft. (**D**) (**a**) Representative fluorescence microscope images of covalently immobilized (RGD-FITC)-decorated Ti-6Al-4V implants before, and 1 and 7 days after surgery (scale bar = 800 μm); (**b**) quantification of the mean fluorescence intensity inferred from the RGD-FITC peptide attached to the surface 1 and 7 days after surgery. Each sample was normalized by their fluorescence intensity before surgery. The green dotted line indicates the fluorescence loss with respect to the fluorescence intensity of the implant before implantation. Data are shown as the means ± the SEM of at least 6 replicas per time point obtained from two independent experiments (unpaired *t*-test).

For the in vivo assessment of the RGD-decorated R-THAB^®^ Ti-6Al-4V implants, we chose to perform a critical size bone defect in mice calvarial. A schematic representation of the surgical procedure and implantation is shown in Figure 7A. The first step consisted of performing the critical size defect; 3 mm proved to be an adequate size for the defect, as it corresponded to the minimum size that was not able to be fully regenerated spontaneously even after 12 weeks (Figure 7B). The following step was the fixation of the insert (Ti-6Al-4V implant + glass coverslip) to the bone. As can be observed in Figure 7C, the Ti-6Al-4V implant was in close contact with the craniotomy’s borders.

To confirm that the peptide layer was still stable after the procedure, we examined the variation on the intensity of RGD-FITC immobilized on the Ti-6Al-4V implants, before and after the surgical intervention. Those results can be seen in Figure 7D, showing that 7 days after surgery, 70% of the RGD-FITC intensity was maintained.

### 3.8. In Vivo Inflammatory Response After Ti-6Al-4V Implantation

**Figure 8:**
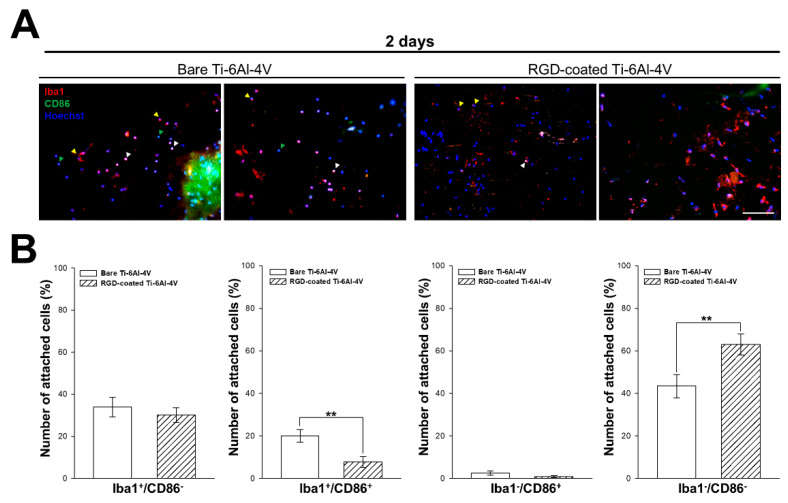
Characterization of the in vivo pro-inflammatory response to bare and RGD-decorated R-THAB^®^ Ti-6Al-4V surfaces 2 days after implantation. (**A**) Representative fluorescence microscopy images of different cell populations on bare and RGD-decorated Ti-6Al-4V surfaces. The macrophage marker Iba1 is stained in red, the CD86 surface marker in green, and the nucleus in blue. Yellow arrows indicate Iba1+/CD86- cells, white arrows cells Iba1+/CD86+, and green arrows Iba1-/CD86+ cells (scale bar = 90 μm). (**B**) Quantification of the percentage of cells from each population on bare and RGD-decorated Ti-6Al-4V implants. Data are shown as the means ± the SEM of 5 samples from 5 mice per condition. The asterisks denote significant differences between bare and RGD-decorated implants (unpaired *t*-test; two asterisks, *p* < 0.01).

To assess the macrophage-related inflammation triggered by Ti-6Al-4V implantation, we performed an analysis to detect pro-inflammatory M1 activated macrophages using a combination of the markers Iba1 (monocytes/macrophage) and CD86 (pro-inflammatory surface marker). The quantification displayed in Figure 8B shows that the number of monocytes/macrophages (Iba1+) detected on bare Ti-6Al-4V implants was higher than on the RGD-decorated surfaces. In addition, a great percentage of them (~20%) were pro-inflammatory M1 macrophages (Iba1+/CD86+), in contrast to the low proportion observed on the RGD-decorated samples (only ~7%). The percentage of Iba1-/CD86+ cells was low in both groups (1–2%), which would correspond to dendritic cells or lymphocytes, and we also observed a great percentage of non-stained cells (Iba1-/CD86-) of unknown lineage. The overall results indicate a reduction in the macrophage-related inflammation motivated by the presence of the RGD layer.

## 4. Discussion

Titanium is commonly used in traumatology as the primary component of devices used for bone replacement or fixation. However, metallic-based prostheses exhibit limited structural and functional integration into the body, which results in progressive instability and the need for repeated surgical interventions throughout a patient’s life. In this context, the development of biomaterials with an enhanced response of the organism may exploit our present knowledge on the Paradigm of Biocompatibility [41]. The Paradigm establishes that the response of the biological system to the biomaterial depends on the recognition by specific cell lineages of the biomolecules (mostly proteins) adsorbed on the material during implantation. Consequently, the Paradigm highlights the importance of the surface of the biomaterial to determine its biocompatibility and, in particular, offers a guide to obtain an intimate contact between the prosthesis and the functional surrounding tissue.

To implement this strategy, numerous biofunctionalization strategies have been proposed over the past few years. However, most of these strategies did not progress to clinical trials. This low rate of success could be attributed to various factors, such as stringent regulations governing research on humans, the complexity of scaling up from laboratory to clinical settings, or simply the lack of reproducibility and control on the procedure. In this work, we demonstrate the potential of peptide-decorated biomaterials by combining R-THAB^®^ Ti-6Al-4V substrates with the EDC/NHS crosslinking chemistry. It is worth highlighting that, although topography and surface roughness may also influence the response of the organism to the implant due to the variation in the number and intensity of the contacts between the cells and the materials, all the experimental campaign was performed on low roughness (rms ≈ 15 μm) samples in order to characterize those effects purely related with the peptide decoration of the samples.

From a wide range of possible candidates of ECM-derived peptides, we selected four oligopeptides for decoration: RGD, CS-1, IKVAV, and PHSRN. The RGD sequence is a cell recognition motif found in fibronectin and other ECM proteins [35], frequently used in combination with various scaffolds in bone regeneration studies [42,43]. CS-1 is a sequence found also in fibronectin and is recognized as a cell adhesion motif [44]. IKVAV is a recognition motif found in laminin, known to have an additive synergistic effect with RGD [35]. It is also known to support in vitro and in vivo adhesion, angiogenesis, and osteogenesis. Lastly, PHSRN is a sequence found in fibronectin that synergizes with RGD to increase cellular activity by activating the α_5_β_1_ integrin [45].

Regarding the long-term stability assays, CS-1 and IKVAV were found to be the most stable peptides. Additionally, their immobilization efficiency surpassed that of RGD or PHSRN. This suggests a potential influence of peptide’s physicochemical properties related to their sequences on immobilization efficiency, as both IKVAV and CS-1 are more hydrophobic than RGD and PHSRN. It should be highlighted, however, that despite these differences, the functionalization procedure allowed the successful immobilization of all peptides on the substrates and a significant stability was observed even after five months. This stability is significative, since the duration should be sufficient for in vivo bone healing, which typically takes 3–4 weeks [2,39].

To analyze the in vitro cellular response induced by each peptide decoration, we employed primary cultures of MSCs on the samples. Polystyrene culture dishes and bare Ti-6Al-4V were used as controls. MSCs were selected for their multipotency and pivotal role in adult bone healing [46,47].

To initially assess the biological response of MSCs to the peptide-decorated R-THAB^®^ Ti-6Al-4V substrates, we evaluated cell adhesion to the decorated material over a 10 h period. With these studies, we aimed to investigate early cell–motif interactions that could influence subsequent stages of cell development (proliferation or differentiation). Among all peptides, RGC, IKVAV, and CS-1 were observed to perform similarly, and any of them outperformed the bare material, but only at later time points (6–10 h). The PHSRN peptide, however, appeared to have minimal impact on cell adhesion.

We also assessed the strength of the cell–substrate interaction on the peptide-decorated Ti-6Al-4V surfaces. This is crucial to ensure that the cells remain firmly adhered to the surfaces. We performed two types of complementary adhesion measurements: (i) single cell detachment using SCFS, and (ii) cell population detachment in response to inverted centrifugation. SCFS provides precise data on the initial interactions between individual cells and the peptides. In contrast, inverted centrifugation assays offer an approximate measure of the bond strength between a fully attached cell population and the peptides. However, Ti-6Al-4V was not suitable for these assays due to technical and safety concerns, so we used borosilicate glass coverslips instead for the inverted centrifugation measurements.

The SCFS measurements indicated that RGD, IKVAV, and CS-1 exhibited superior performance compared to the bare surface, suggesting strong specific ligand–integrin interactions. In contrast PHSRN showed a lesser effect, although it still outperformed the bare material. These results are consistent with comparable experiments found in the literature. Mardilovich et al. [48] used AFM to directly measure the interaction between integrin α_5_β_1_ and monolayers of RGD and PHSRN. Their results indicated a stronger interaction with RGD compared to PHSRN, although PHSRN still exhibited a higher binding affinity than a scrambled peptide control.

Inverted centrifugation studies demonstrated that cells seeded on RGD-, CS-1-, and IKVAV-decorated glass substrates were more resistant to detachment during centrifugation compared to those on bare or on the PHSRN-decorated material, in agreement with the SCFS results. In this context, and while the PHSRN peptide can interact with cells and seemed to slightly improve initial adhesion strength, it seemed unable to withstand high centrifugal forces, possibly due to lower surface concentration. This highlights the specificity of peptide-mediated cell interactions and the importance of selecting appropriate ligands to enhance cell behavior.

Based on its global in vitro performance, we decided to choose RGD as our preferred peptide for continuing the experimental campaign. In addition, RGD is a well-established peptide for enhancing biomaterial osseointegration due to its ability to bind multiple integrins, including α_5_β_1_, α_V_β_5_, and α_V_β_3_, which play crucial roles in bone regeneration in vivo [45]. However, prior to in vivo studies, we assessed both the proliferation/survival and osteogenic differentiation of MSCs seeded on Ti-6Al-4V substrates. Our results showed how RGD decoration enhanced the proliferation rate of MSCs on Ti-6Al-4V surfaces, enabling them to achieve similar expansion rates to those observed on polystyrene dishes (control in vitro condition). While RGD decoration enhanced MSC proliferation on Ti-6Al-4V, the overall cell number remained lower than on polystyrene dishes. This difference likely stems from the reduced initial adhesion observed on Ti-6Al-4V compared to polystyrene during the 2–10 h period. Positive results were also obtained from RGD-decorated substrates when assessing the differentiation of MSCs on the different substrates. It was observed that RGD-decorated Ti-6Al-4V surfaces enhanced osteogenic differentiation compared not only to bare Ti-6Al-4V substrates, but also to polystyrene controls. This effect was only observed in samples incubated with osteogenic differentiation factors, confirming that RGD alone does not induce differentiation. The ability of the RGD peptide to promote osteogenic differentiation has been widely described in the literature [49,50]. These outstanding results demonstrated that the RGD decoration can significantly enhance cell behavior on R-THAB^®^ Ti-6Al-4V, surpassing even standard polystyrene culture dishes, particularly after cells become fully attached.

The in vitro characterization of the peptide-decorated substrates was complemented with an in vivo study, whose primary goals were to assess the viability of the implantation procedure, and the in vivo impact of the RGD decoration on the response to the implant. Among the possible models for studying in vivo bone formation on implanted biomaterials, we chose the calvarial model because of its simplicity. The calvarial defect model is commonly used to assess bone growth on hydrogels [42,51] or 3D-printed polymeric scaffolds [52], so we developed an innovative procedure for implanting solid metallic implants.

To develop our calvarial defect model, we reviewed the literature to identify the most commonly used critical-sized defects in mice. We found that 3 to 4 mm diameter defects were the most prevalent [53,54] and confirmed that the 3 mm model was suitable for our study. For the implantation, we prepared circular 3 mm diameter R-THAB^®^ Ti-6Al-4V samples, which were decorated using our established protocol. Considering the parietal bone thickness in adult mice (35–40 g) to be approximately 150–250 μm [55], we set the implant thickness to 200 μm. The 5 mm glass coverslips used in combination with the Ti-6Al-4V implants facilitated their handling and allowed their fixation to the surrounding bone. This in vivo model supported the stability of the functionalized layer, as the FITC-labeled RGD decoration retained 70% of its fluorescence for a week.

To assess the initial in vivo effect of the RGD decoration, we focused on studying the inflammatory response, since it corresponds to the first stage in any post-surgical healing [25,56]. While it is not definitively proven that inflammation is the primary cause of implant encapsulation, it is well established that it is linked to fibrosis development [57]. To simplify this assessment, we focused on studying the presence of macrophage-related inflammation among the cells surrounding the implant. However, this characterization was limited by several factors, including the presence of native tissue on the extracted implants, which could lead to non-specific staining, and the clustering of cells, making individual analysis difficult. Despite these limitations, we observed a significant reduction in macrophage presence and pro-inflammatory activation on RGD-decorated implants. While the exact mechanism of RGD’s influence on inflammation remains unclear, some studies suggest that it has an immunoregulatory effect. For instance, Guo et al. [58] demonstrated that dopamine-RGD coatings on titanium significantly reduced inflammation in both in vivo and in vitro models. In this article, the dopamine-RGD coatings on titanium reduced the infiltration of both CD68^+^ macrophages and pro-inflammatory CD68^+^/CD86^+^ macrophages into the *peri*-implant area. Our results, obtained from the implant surface, are consistent with these findings.

At this point, it is worth highlighting that the main result of this work may be regarded as simply the validation of the biomaterial decoration strategy as a more than promising approach for the development of implants with enhanced biological response. It is evident, however, that the full exploitation of this strategy will require finding answers to such pressing questions as the identification of the membrane proteins that interact with the peptides, the determination of the optimal conditions for the differentiation of the cells to osteoblasts both in vitro and in vivo, and the development of a surgical procedure that promotes the osseointegration of the decorated materials, just to mention a few. It is evident that the answer to all these questions can only be the result of intensive future work.

## 5. Conclusions

In this work, we studied the decoration of R-THAB^®^ Ti-6Al-4V samples with peptides in order to establish a strategy that may lead to implants with enhanced biological response. The performance of the decorated materials was characterized in vitro and in vivo on four selected peptides from extracellular matrix proteins. As a result of the in vitro tests on these peptides, the RGD peptide was selected to proceed with the characterization of the proliferation and osteogenic differentiation of MSCs on the material. We observed an enhancement on MSC proliferation and osteogenic differentiation on the decorated substrates in comparison with the bare Ti-6Al-4V material. In addition, the application of these RGD-decorated Ti-6Al-4V substrates in a calvarial model in vivo leads to a reduction in the initial macrophage-related inflammation stimulated by the implantation procedure.

In summary, our results show that the combination of peptide decoration on R-THAB^®^ functionalized surfaces represents a promising strategy for the obtaining of metallic biomaterials with enhanced biocompatibility that may contribute to the progress of bone tissue replacement therapies for skeletal disorders in the future.

## Figures and Tables

**Table 1:** Summary of RGD, CS-1, IKVAV, and PHSRN performance during the in vitro cellular behavior assessments. The asterisks correspond to the statistical differences (one asterisk, *p* < 0.05; two asterisks, *p* < 0.01) obtained for every peptide-decorated material when compared to the bare material in each study. Adhesion kinetics results are gathered from decorated-Ti-6Al-4V samples, whereas adhesion strength corresponds to results obtained from decorated glass samples.

			Adhesion Strength		
	Adhesion Kinetics		SCFS	Inverted Centrifugation	
RGD	2 h	*	**	1 RCF	*
	6 h	**		8 RCF	*
CS-1	2 h	-	**	1 RCF	-
	6 h	*		8 RCF	*
IKVAV	2 h	-	**	1 RCF	*
	6 h	*		8 RCF	-
PHSRN	2 h	-	*	1 RCF	-
	6 h	-		8 RCF	-

## Data Availability

Data may be available upon reasonable request to the corresponding authors.
